# One-year outcomes of a single bolus r-SAK before primary PCI for STEMI: Follow-up of the OPTIMA-5 study

**DOI:** 10.7555/JBR.39.20250043

**Published:** 2025-05-21

**Authors:** Chen Li, Jie Yu, Tian Wu, Qingxia Lin, Rui Hua, Zihang Zhong, Yule Li, Kun Liu, Li Zhu, Naiquan Yang, Xin Chen, Xiaoyan Wang, Xin Zhao, Jun Jiang, Bo Zhao, Xiwen Zhang, Pengsheng Chen, Tong Wang, Yi Xu, Gaoyong Liao, Liang Yuan, Bo Chen, Zhihui Xu, Xiaoxuan Gong, Wenhao Zhang, Chunyue Tan, Lei Xu, Qiang Huang, Jianling Bai, John W. Eikelboom, Chunjian Li

**Affiliations:** 1 Department of Cardiology, the First Affiliated Hospital of Nanjing Medical University, Nanjing, Jiangsu 210029, China; 2 Department of Biostatistics, School of Public Health, Nanjing Medical University, Nanjing, Jiangsu 211166, China; 3 College of Letters and Science, University of Wisconsin−Madison, Madison, WI 53715-1007, USA; 4 Department of Cardiology, the First People's Hospital of Lianyungang, Lianyungang, Jiangsu 222002, China; 5 Department of Cardiology, Taizhou People's Hospital, Taizhou, Jiangsu 225300, China; 6 Department of Cardiology, Huai'an Second People's Hospital Affiliated to Xuzhou Medical University, Huai'an, Jiangsu 223200, China; 7 Department of Cardiology, the Affiliated Changzhou No. 2 People's Hospital of Nanjing Medical University, Changzhou, Jiangsu 213164, China; 8 Department of Cardiology, Affiliated Hospital of Jiangnan University, Wuxi, Jiangsu 214000, China; 9 Department of Cardiology, the Second Hospital of Dalian Medical University, Dalian, Liaoning 116027, China; 10 Department of Cardiology, the Second Affiliated Hospital of Zhejiang University School of Medicine, Hangzhou, Zhejiang 310009, China; 11 Department of Cardiology, the Affiliated Huai'an No. 1 People's Hospital of Nanjing Medical University, Huai'an, Jiangsu 223300, China; 12 Department of Cardiology, Xuzhou Central Hospital, Xuzhou, Jiangsu 221000, China; 13 Department of Cardiology, Yancheng No. 1 People's Hospital, Yancheng, Jiangsu 224000, China; 14 Department of Radiology, the First Affiliated Hospital of Nanjing Medical University, Nanjing, Jiangsu 210029, China; 15 Xintrum Pharmaceuticals Co., Ltd., Nanjing, Jiangsu 211100, China; 16 Department of Medicine, McMaster University or Thrombosis Service, Hamilton General Hospital, Hamilton, Ontario L8S 4L8, Canada

**Keywords:** recombinant staphylokinase, myocardial infarction, thrombolysis, percutaneous coronary intervention

## Abstract

The Optimal Management of Antithrombotic and Thrombolytic Agents-5 (OPTIMA-5) study demonstrated that a single bolus of half the standard dose of recombinant staphylokinase (r-SAK) before primary percutaneous coronary intervention (PCI) significantly improved the patency of the infarct-related artery in patients with ST-segment elevation myocardial infarction (STEMI), who were expected to undergo PCI within 120 min. The present study aimed to investigate the one-year clinical outcomes and the effect of the anti-r-SAK antibodies on a second r-SAK thrombolysis in OPTIMA-5 patients. The clinical outcome measured was major adverse cardiovascular events (MACE) within 360 days. Patients' anti-r-SAK antibody levels were determined on day 90 (± 7 days), day 180 (± 7 days), and day 360 (± 14 days) after thrombolysis, and *in vitro* r-SAK antibody neutralization experiments were performed to explore an optimal interval for a second r-SAK thrombolysis. Results showed that the MACE incidence was numerically lower in the r-SAK group compared with the normal saline (NS) group (14.0% *vs*. 20.0%, hazard ratio [HR] = 0.67, 95% confidence interval [CI]: 0.34–1.32; log-rank *P* = 0.245). The anti-r-SAK antibody levels in the r-SAK group decreased with time, but remained significantly higher than those in the NS group on day 90 (± 7 days) (2.96 ± 0.68 *vs*. 0.22 ± 0.53, *P* < 0.001), day 180 (± 7 days) (2.19 ± 0.74 *vs*. 0.44 ± 0.65, *P* < 0.001), and day 360 (± 14 days) (1.73 ± 0.97 *vs*. 0.37 ± 0.71, *P* < 0.001). The *in vitro* anti-r-SAK antibody neutralization experiments demonstrated that the thrombolysis rate decreased exponentially as the antibody titer increased from 1.90 to 2.20 (67.80% ± 14.19% *vs*. 44.32% ± 21.54%, *P* < 0.0001). Therefore, for STEMI patients who are expected to undergo PCI within 120 min, a single bolus of half-dose r-SAK before primary PCI may reduce the one-year MACE risk. The anti-r-SAK antibody persists over one year, and a second r-SAK thrombolysis may not be indicated until at least one year after the first administration, if necessary.

## Introduction

ST-segment elevation myocardial infarction (STEMI) is the leading cause of cardiovascular mortality worldwide, characterized by complete coronary artery occlusion primarily because of acute thrombosis^[1]^. Rapid reperfusion with primary percutaneous coronary intervention (PCI) within 120 min of first medical contact is currently recommended by clinical guidelines, while thrombolytic therapy is only recommended when primary PCI is expected to be delayed by more than 120 min after presentation ("pharmacoinvasive PCI")^[2–3]^.

The Optimal Management of Antithrombotic and Thrombolytic Agents-5 (OPTIMA-5) study investigated whether adjunctive thrombolysis benefits STEMI patients who are expected to receive PCI within 120 min of presentation^[4]^. The study first adopted a highly fibrin-specific, third-generation thrombolytic agent, recombinant staphylokinase (r-SAK), in combination with contemporary antithrombotic agents ticagrelor and aspirin, followed by immediate PCI ("contemporary facilitated PCI"), and demonstrated that a single bolus of half-dose r-SAK compared with normal saline (NS) before primary PCI provided a higher rate of thrombolysis in myocardial infarction (TIMI) flow grade 2 to 3, along with a reduction in infarct size, without an increase in major bleeding^[4]^.

However, two major issues remain a concern in the OPTIMA-5 study. (1) Though r-SAK thrombolysis was associated with increased patency and reduced infarct size, no improvement was observed in predefined 360-day major adverse cardiovascular and cerebrovascular events (MACCE) (r-SAK 11.0% *vs*. NS 10.0%, hazard ratio [HR] = 1.10, 95% confidence interval [CI]: 0.47–2.58, *P* = 0.833)^[4]^. Considering that heart failure and cardiogenic shock are important adverse cardiovascular events after STEMI that were not included in the previously defined MACCE, it is necessary to refine the definition and reanalyze the difference in the adjusted major adverse cardiovascular events (MACE) between groups. (2) Staphylokinase (SAK) is produced by *Staphylococcus aureus*, and r-SAK is a recombinant form of SAK, which is a heterologous protein and may cause an immune response. The long-term dynamic changes in the antibody levels against r-SAK after thrombolysis, as well as whether antibody levels will affect a second r-SAK thrombolysis, remain unclear. Accordingly, the present study aimed to investigate the one-year clinical outcomes and the effect of the anti-r-SAK antibodies on a second r-SAK thrombolysis in patients from the OPTIMA-5 study.

## Subjects and methods

This study was registered at ClinicalTrials.gov (NCT05649696).

### Study design

**Figure 1:**
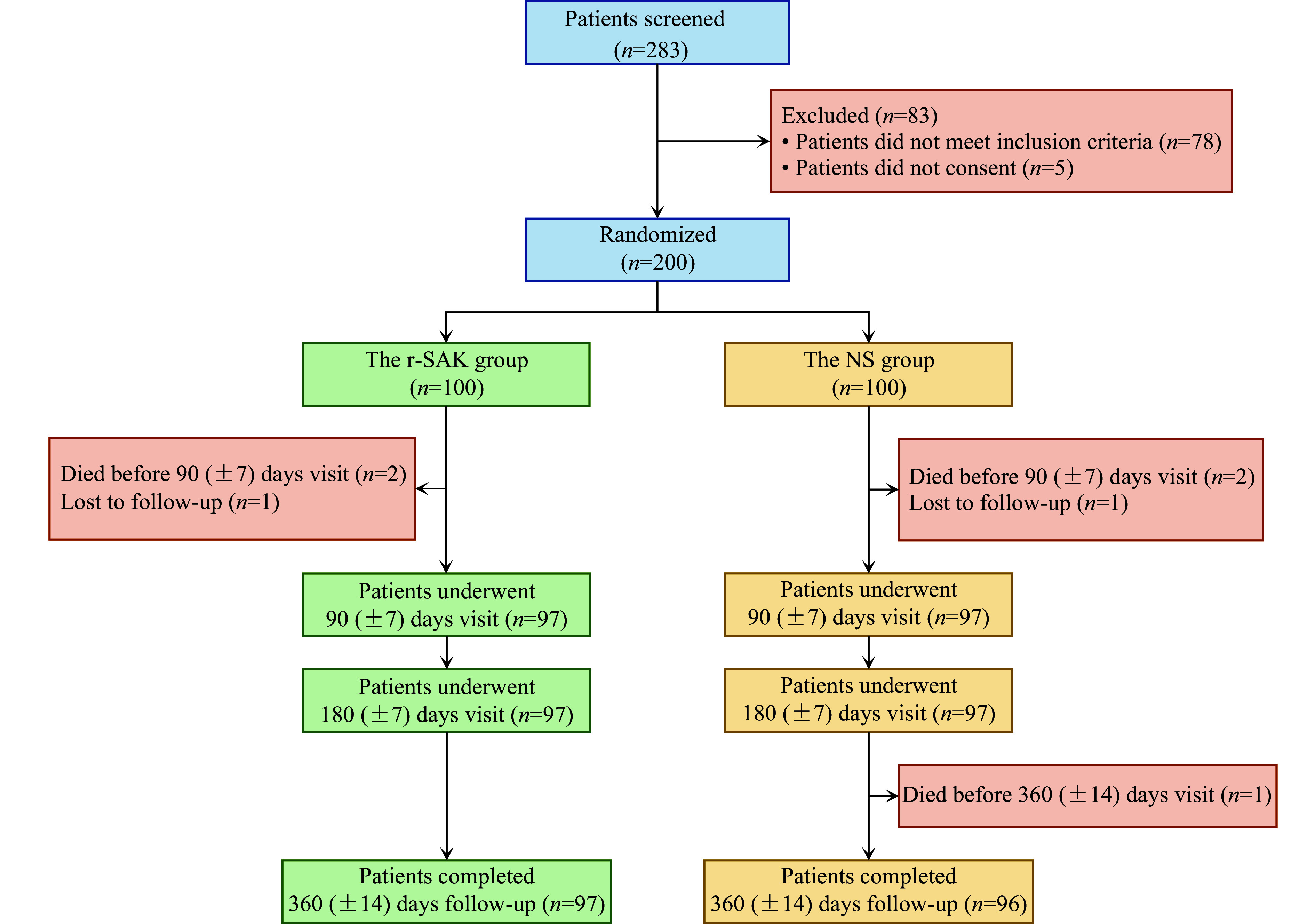
Study flow chart. Abbreviations: NS, normal saline; r-SAK, recombinant staphylokinase.

The OPTIMA-5 study (NCT05023681) was an investigator-initiated, prospective, multicenter, randomized, controlled trial comparing a single bolus of half-dose r-SAK with normal saline (NS) in patients with STEMI presenting within 12 h of symptom onset and expected to undergo PCI within 120 min of presentation (***Fig. 1***)^[4]^.

The OPTIMA-5 Steering Committee and investigators (***Supplementary Data 1***, available online) conceived, designed, and conducted the one-year follow-up of the OPTIMA-5 study. This study protocol, which adhered to the Declaration of Helsinki and Good Clinical Practice guidelines, was approved by the Institutional Review Board of the First Affiliated Hospital of Nanjing Medical University (approval number 2021-SR-309). Written informed consent was obtained from all patients before their inclusion in the study.

### Study protocol

STEMI patients aged 18–75 years, weighing ≥ 45 kg, with a chest pain onset time of ≤ 12 h and estimated primary PCI time of ≤ 120 min, were enrolled in the OPTIMA-5 study^[4]^. All recruited patients received a loading dose of 300 mg aspirin, 180 mg ticagrelor, and an intravenous bolus of unfractionated heparin (60 U/kg and up to a maximum of 5000 U). Patients were randomized to either the r-SAK or the control group. Those assigned to the r-SAK group received an intravenous bolus of 5 mg r-SAK administered over three minutes before undergoing coronary arteriography (CAG), whereas those assigned to the control group received NS over the same time span.

CAG was performed at 15 frames per second, lasting for at least five cardiac cycles or until the contrast agent was fully drained, with specific projections based on the location of the infarct-related artery (IRA)^[4]^. Immediate PCI was mandated if the TIMI flow of the IRA was graded 0–2. However, the PCI strategy was left to the discretion of the cardiologists if the TIMI flow reached grade 3. The use of platelet glycoprotein Ⅱb/Ⅲa receptor inhibitor (GPI) or low-molecular-weight heparin (LMWH) was not recommended unless there was evidence of heavy thrombus burden or no/slow reflow in CAG.

### Clinical outcomes

Patients were followed up on day 90 (± 7 days), day 180 (± 7 days), and day 360 (± 14 days) after randomization, during which adverse cardiovascular events were recorded.

The primary clinical outcome was defined as MACE within 360 days, including all-cause death, reinfarction, unplanned target vessel revascularization (TVR), heart failure or cardiogenic shock, and major ventricular arrhythmia. The secondary clinical outcomes included each of the above MACE events, cardiovascular death, cardiac mechanical complications (including ventricular septal rupture, papillary muscle rupture, cardiac rupture, and ventricular aneurysm), and stroke within 360 days. MACE events were adjudicated by an independent event review committee (ERC) based on prespecified definitions (***Supplementary Data 2***, available online). Committee members were blinded to treatment allocation to ensure an unbiased evaluation of the clinical outcomes.

MACE was evaluated in subgroup analyses according to baseline characteristics, including age, sex, Killip class, hypertension, diabetes, prior intervention, smoking status, infarct location, IRA, and time from symptom onset to r-SAK or NS infusion.

### Detection of the anti-r-SAK antibody

Venous blood samples (4 mL) were collected in serum separator tubes (BD Vacutainer, Franklin Lakes, NJ, USA) at every clinical follow-up visit. The blood samples were centrifuged at 2000 *g* for 15 min at 2–8 ℃, and the supernatants were transferred into cryogenic vials (Servicebio, Wuhan, China). The serum samples were transported under cold-chain conditions at −80 ℃ to Xintrum Pharmaceuticals Co., Ltd. (Nanjing, Jiangsu, China) for anti-r-SAK antibody testing using the enzyme-linked immunosorbent assay (ELISA; ***Supplementary Data 3***, available online).

### *In vitro* anti-r-SAK antibody neutralization experiments

Ten patients were enrolled to provide blood samples for clot formation. The inclusion criteria were as follows: (1) Age 18–75 years and weight ≥ 45 kg; (2) Hospitalized patients with suspected coronary artery disease, who were scheduled for CAG or intervention; (3) Patients who had received loading doses of 300 mg aspirin and 180 mg ticagrelor, or maintenance doses of these drugs for more than three days. Patients previously treated with r-SAK thrombolysis or those with a confirmed history of *Staphylococcus aureus* infection were excluded.

**Figure 2:**
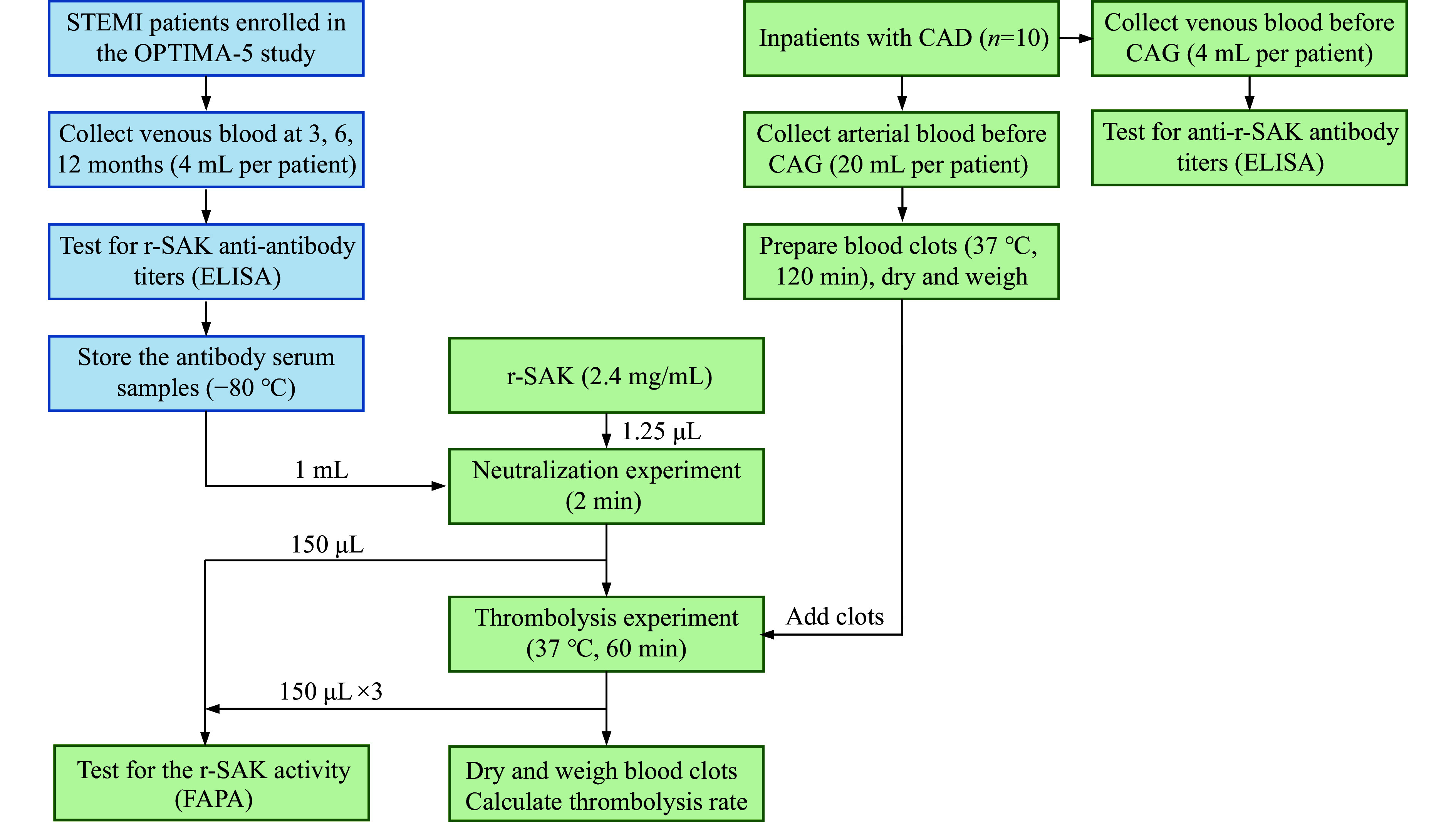
Procedures of *in vitro* thrombolysis experiments. Abbreviations: CAD, coronary artery disease; CAG, coronary angiography; ELISA, enzyme-linked immunosorbent assay; FAPA, fibrin-agarose plate assay; OPTIMA-5, Optimal Management of Antithrombotic and Thrombolytic Agents-5 (OPTIMA-5); r-SAK, recombinant staphylokinase; STEMI, ST-elevation myocardial infarction.

The process of *in vitro* anti-r-SAK antibody neutralization experiments is illustrated in ***Fig. 2***. Venous blood samples (4 mL) were collected into centrifuge tubes from each recruited patient to determine the baseline anti-r-SAK antibody levels. Subsequently, 20 mL of arterial blood was collected from each patient and equally distributed into cryogenic vials. The vials were incubated at 37 ℃ for 120 min to induce blood clot formation. The resulting blood clots were collected, washed with NS, dried on gauze, and weighed using a digital balance (TLE204E, Mettler Toledo, Columbus, OH, USA).

Neutralization experiments were performed by mixing 1 mL of the serum from OPTIMA-5 patients with varying anti-r-SAK antibody titers and 1.25 μL of r-SAK (final concentration of r-SAK: 0.003 mg/mL). Two minutes after mixing, 150 μL of the serum mixture was transferred to a centrifuge tube (Biosharp, Beijing, China) and stored at −80 ℃ for the measurement of pre-thrombolysis r-SAK activity using the fibrin-agarose plate assay (FAPA; ***Supplementary Data 4***, available online).

The prepared blood clots were then added individually to each mixture and incubated at 37 ℃ for 60 min. The supernatants were transferred into centrifuge tubes (150 μL per tube) and stored at −80 ℃ for the measurement of post-thrombolysis r-SAK activity using FAPA. The remaining blood clots were washed with NS, dried on gauze, and reweighed. The thrombolysis rate was calculated as follows: thrombolysis rate (%) = [(initial clot weight − final clot weight) / initial clot weight] × 100%.

### Detection indicators

The experimental detection indicators included: (1) anti-r-SAK antibody levels in OPTIMA-5 patients (day 90 [± 7 days], day 180 [± 7 days], and day 360 [± 14 days]); (2) *in vitro* thrombolysis rate after mixing with different anti-r-SAK antibody titers; and (3) r-SAK activity before and after *in vitro* thrombolysis.

### Statistical analysis

Continuous variables were presented as mean ± standard deviation or median (interquartile range) and compared between groups using the Student's *t*-test or Mann–Whitney *U* test, as appropriate. Categorical variables were presented as frequencies (percentages) and compared using the Chi-square test or Fisher's exact test. Thrombolysis rates and r-SAK activity, grouped by antibody titers, were compared using one-way ANOVA, with pairwise comparisons conducted using the least significant difference test. A nonlinear regression model with a logit transformation of thrombolysis rates was constructed to estimate the relationship between the anti-r-SAK antibody titers and thrombolysis rates. Bartlett's test and robust Levene's test were used to assess homoscedasticity, and the weighted least squares method was applied to correct for heteroscedasticity. The Kaplan–Meier curve, evaluated using the log-rank test, was used to evaluate the one-year MACE incidence. Subgroup analyses were performed using Cox proportional hazards regression model, with results presented as HRs and 95% CIs. All patients enrolled in the OPTIMA-5 study were included in the analyses according to the intention-to-treat principle. Patients lost to follow-up were censored at the time of their last known alive status. A two-sided *P* value < 0.05 was considered statistically significant. All statistical analyses were performed using SPSS Statistics (version 26.0; IBM, Armonk, NY, USA) and GraphPad Prism (version 9.0; GraphPad Software, San Diego, CA, USA).

## Results

### Baseline characteristics

Between October 29, 2021, and August 14, 2022, a total of 200 patients were recruited from eight sites across China. The demographic and interventional characteristics of the study patients have been previously reported in the OPTIMA-5 study^[4]^. The Synergy Between PCI with Taxus and Cardiac Surgery (SYNTAX) scores in the r-SAK and NS groups were comparable [11.5 (8.0–17.9) *vs.* 14.3 (8.8–20.6); *P* = 0.105], indicating that the baseline complexity of coronary artery lesions between the two groups was well-balanced.

### Clinical outcomes

**Figure 3:**
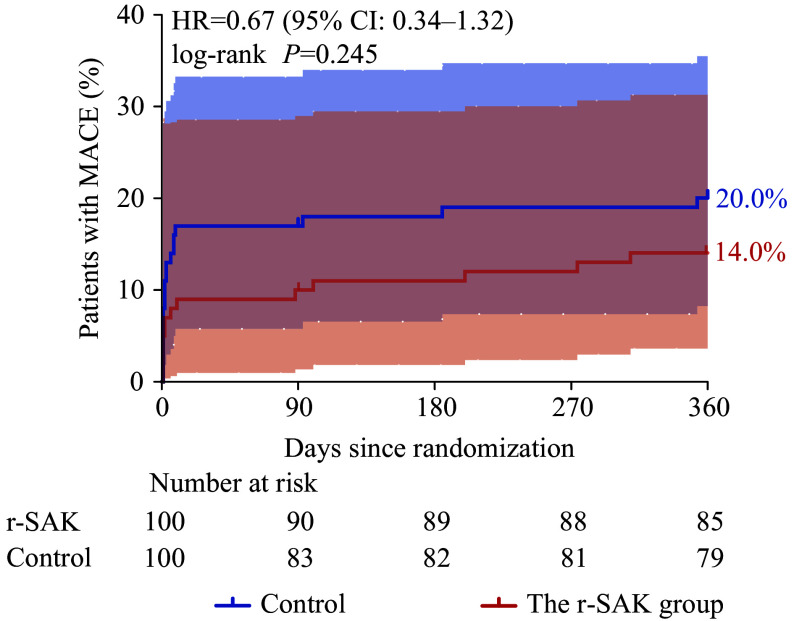
Clinical outcomes of the r-SAK group *vs.* the NS group in OPTIMA-5 patients. The Kaplan–Meier curve (tested with the log-rank test) was used to evaluate the 1-year MACE incidence. The incidence of 1-year MACE in the r-SAK group was numerically lower than that in the NS group. Abbreviations: CI, confidence interval; HR, hazard ratio; MACE, major adverse cardiovascular events; NS, normal saline; r-SAK, recombinant staphylokinase.

During the follow-up period, five patients died, two patients were lost to follow-up, and 193 patients completed the one-year clinical follow-up (***Fig. 1***). The incidence of MACE was lower in the r-SAK group compared with the NS group (14.0% *vs*. 20.0%, HR = 0.67, 95% CI: 0.34–1.32; log-rank *P* = 0.245), although the difference did not reach statistical significance (***Fig. 3***). Two patients (2.0%) in the r-SAK group and three (3.0%) in the NS group died. Except for one death caused by intestinal cancer in the NS group, all the other deaths were attributed to cardiovascular causes (three cardiogenic shocks and one ventricular septal rupture). The incidence rates of reinfarction (2.0% *vs*. 1.0%; *P* = 1.000), unplanned unplanned target vessel revascularization (TVR) (1.0% *vs*. 2.0%; *P* = 1.000), heart failure or cardiogenic shock (10.0% *vs*. 18.0%; *P* = 0.103), cardiovascular death (2.0% *vs*. 2.0%; *P* = 1.000), cardiac mechanical complications (8.0% *vs*. 10.0%; *P* = 0.621), and stroke (2.0% *vs*. 2.0%; *P* = 1.000) showed no statistically significant differences between the groups. No major ventricular arrhythmias were observed during the follow-up period. In the r-SAK group, there were nine cases of heart failure and three cases of cardiogenic shock; while in the NS group, there were 17 cases of heart failure and three cases of cardiogenic shock. The cardiac mechanical complications in the r-SAK group included seven cases of ventricular aneurysms and one case of ventricular septal rupture, whereas the NS group had eight ventricular aneurysms and two papillary muscle ruptures (***Table 1***).

**Table 1:** Clinical outcomes at 360-day follow-up

Clinical outcomes	The r-SAK group (*n*=100)	The NS group (*n*=100)	*P*-value
MACE	14 (14.0)	20 (20.0)	0.259
All-cause death	2 (2.0)	3 (3.0)	1.000
Reinfarction	2 (2.0)	1 (1.0)	1.000
Unplanned TVR	1 (1.0)	2 (2.0)	1.000
Heart failure or cardiogenic shock	10 (10.0)	18 (18.0)	0.103
Heart failure	9 (9.0)	17 (17.0)	0.093
Cardiogenic shock	3 (3.0)	3 (3.0)	1.000
Major ventricular arrhythmia	0 (0)	0 (0)	NA
Cardiovascular death	2 (2.0)	2 (2.0)	1.000
Cardiac mechanical complications	8 (8.0)	10 (10.0)	0.621
Ventricular septal rupture	0 (0)	0 (0)	NA
Papillary muscle rupture	0 (0)	2 (2.0)	0.497
Cardiac rupture	1 (1.0)	0 (0)	1.000
Ventricular aneurysm	7 (7.0)	8 (8.0)	1.000
Stroke	2 (2.0)	2 (2.0)	1.000
The categorical variables are presented as counts (percentages) and compared using the *χ*^2^ test between the two groups. In terms of MACE, in the r-SAK group, one patient experienced both heart failure or cardiogenic shock and all-cause death was counted as one MACE. In the NS group, two patients with both heart failure or cardiogenic shock and all-cause death were counted as two MACEs, and one patient with heart failure or cardiogenic shock, reinfarction, and unplanned TVR was counted as one MACE. Regarding heart failure or cardiogenic shock, two patients in both the r-SAK group and the NS group experienced concurrent heart failure and cardiogenic shock. Abbreviations: MACE, major adverse cardiovascular events; NA, not applicable; NS, normal saline; r-SAK, recombinant staphylokinase; TVR, target vessel revascularization.			

**Figure 4:**
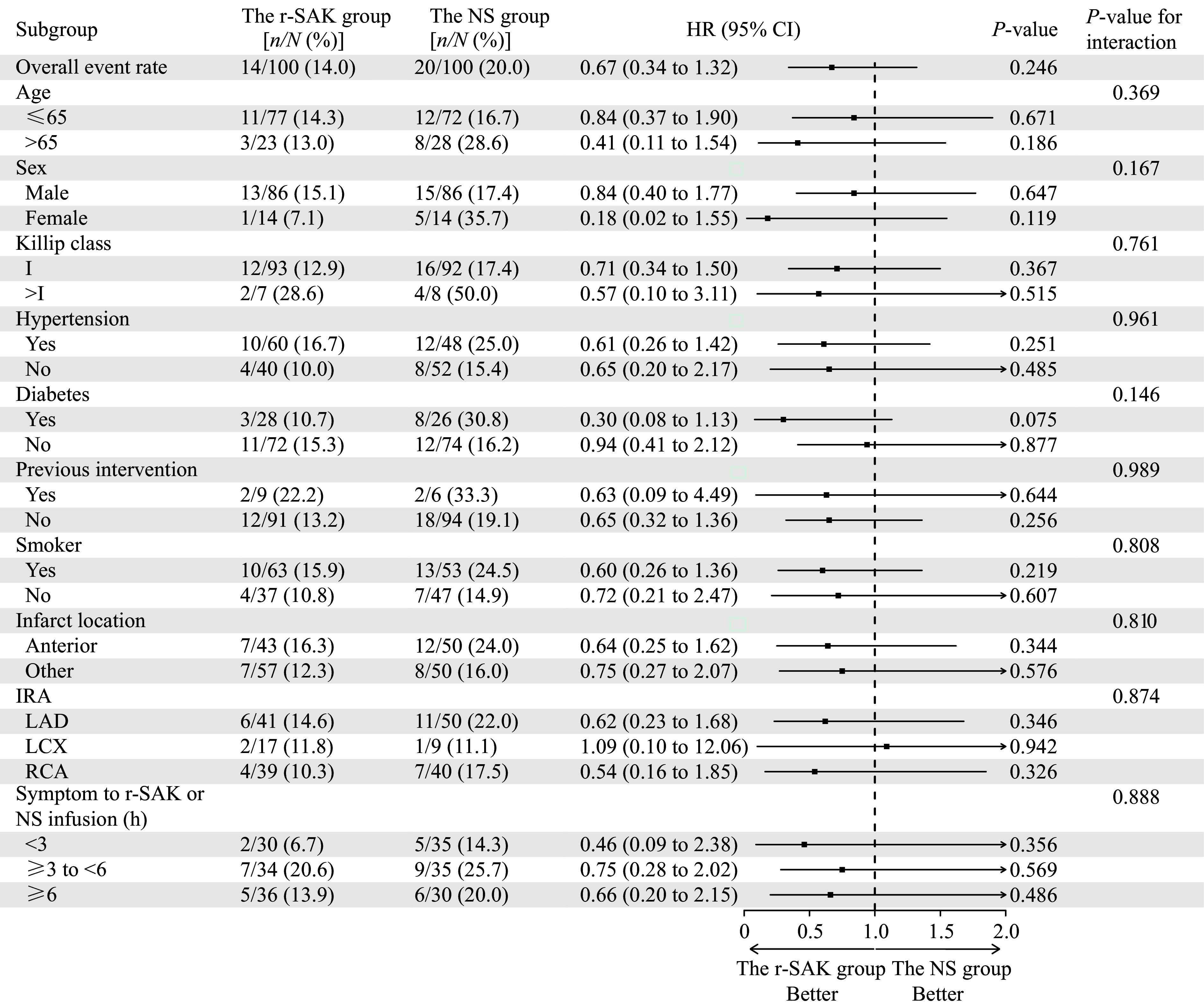
Major adverse cardiovascular events in subgroup analysis. Subgroup analysis was performed using Cox proportional hazards regression, with results presented as HRs and 95% CIs. Abbreviations: HR, hazard ratio; CI, confidence interval; IRA, infarct-related artery; LAD, left anterior descending artery; LCX, left circumflex artery; *n* and *N* indicate the number of events and patients, respectively; NS, normal saline; RCA, right coronary artery; r-SAK, recombinant staphylokinase.

The subgroup analysis of MACE incidence is shown in ***Fig. 4***. The MACE rate was consistently lower with the contemporary facilitated PCI strategy across all subgroups, except when the IRA was the left circumflex artery.

### Experimental results

**Figure 5:**
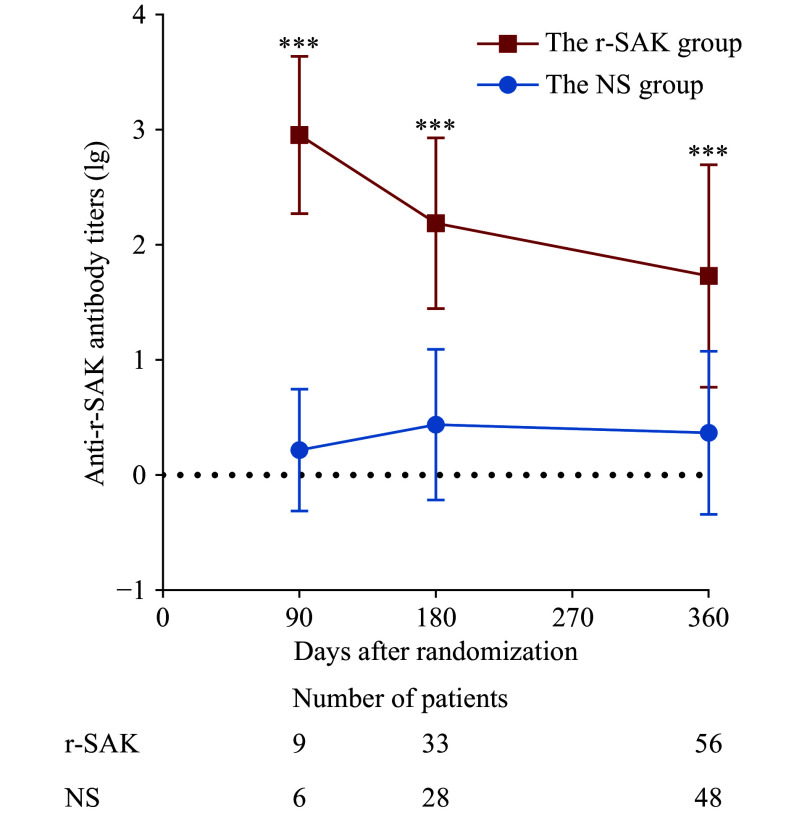
Variations of anti-r-SAK antibody levels in OPTIMA-5 patients. The comparisons of anti-r-SAK antibody titers between groups at each visit were performed using the unpaired *t*-test with Welch correction. ^***^P < 0.001 compared with the NS group. Abbreviations: NS, normal saline; r-SAK, recombinant staphylokinase.

The anti-r-SAK antibody titers (lg) in the r-SAK group decreased over time but remained significantly higher than those in the NS group on day 90 (2.96 ± 0.68 *vs*. 0.22 ± 0.53, *P* < 0.001), day 180 (2.19 ± 0.74 *vs*. 0.44 ± 0.65, *P* < 0.001), and day 360 (1.73 ± 0.97 *vs*. 0.37 ± 0.71, *P* < 0.001) (***Fig. 5***). The anti-r-SAK antibody was detected in 25.6% of all samples from the NS group, with a titer (lg) of 0.38 (± 0.67).

Among the 10 patients recruited for the neutralization experiments, one exhibited an anti-r-SAK antibody titer (lg) of 1.60, another had a titer below 1.30, and the remaining eight patients showed baseline anti-r-SAK antibody titers (lg) of zero.

**Figure 6:**
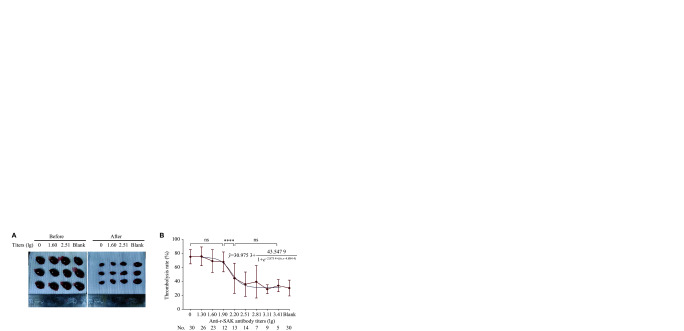
*In vitro* thrombolysis rates at different anti-r-SAK antibody titers. A: Blood clots before and after *in vitro* thrombolysis. B: Variations of *in vitro* thrombolysis rates at different anti-r-SAK antibody titers. Thrombolysis rates grouped by antibody titers were compared using one-way ANOVA, and pairwise comparisons were performed using the least significant difference test method. The nonlinear regression model utilizing a logit transformation of thrombolysis rates was constructed to estimate the relationship between the anti-r-SAK antibody titers and thrombolysis rates. Bartlett's test and robust Levene's test were used to assess homoscedasticity, and the weighted least squares method was applied to account for heteroscedasticity. ^****^P < 0.0001. Abbreviations: ns, no significance; r-SAK, recombinant staphylokinase; No., number of patients.

In the antibody-negative control group, the thrombolysis rate was 75.17% (± 10.18%), showing a non-significant decrease (*P* = 0.134) as the antibody titer (lg) increased to 1.90. Subsequently, the thrombolysis rate significantly decreased to 44.32% (± 21.54%) (*P* < 0.0001) as the antibody titer (lg) increased to 2.20 (***Fig. 6A*** and ***6B***). The relationship between the thrombolysis rate (*y*) and the anti-r-SAK antibody titer (*x*) followed an exponential pattern, as shown in ***Fig. 6B***.

**Figure 7:**
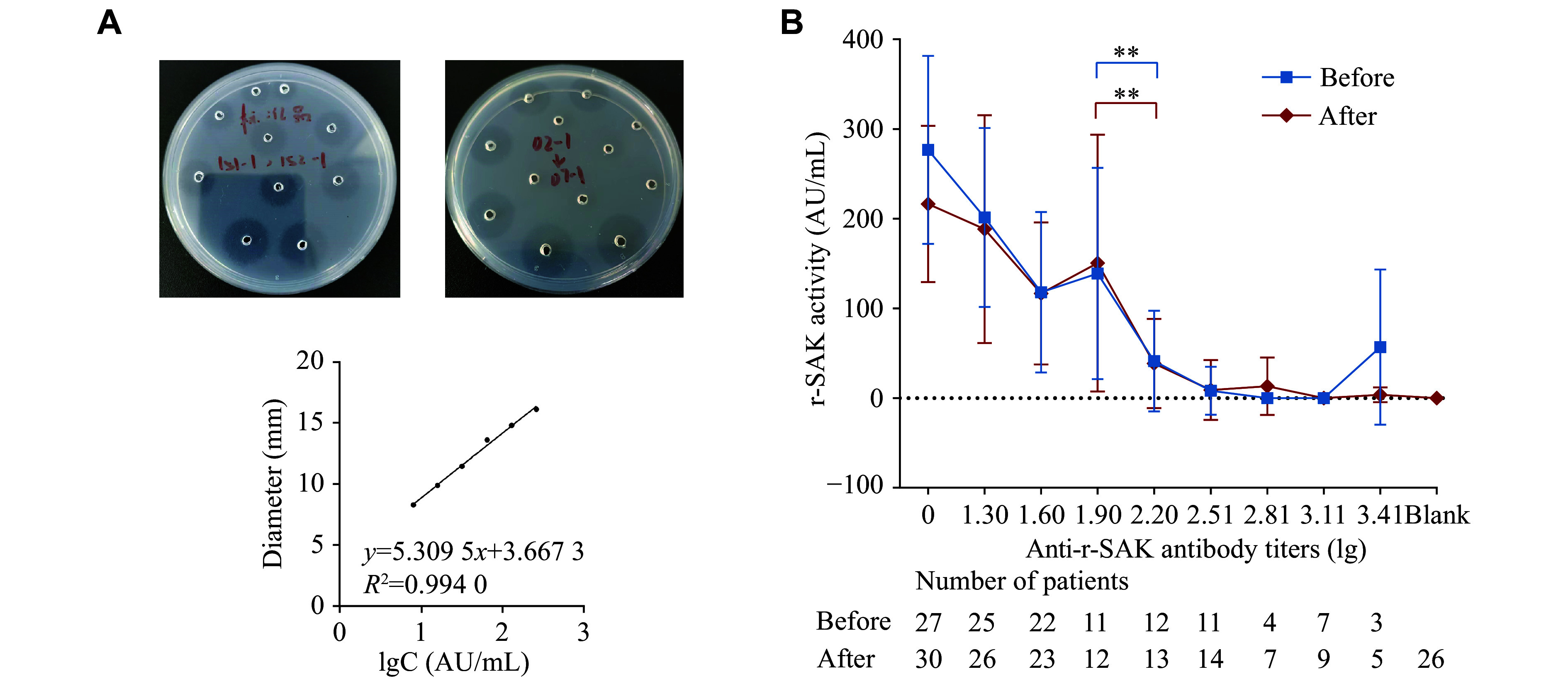
Determination of r-SAK activity before and after *in vitro* thrombolysis. A: Images from the FAPA experiment, showing a standard well plate (left) and an experimental well plate (right). Diameter-log concentration curve (Diameter-lgC curve) was used for the quantitative analysis of r-SAK in FAPA. B: r-SAK activity at different anti-r-SAK antibody titers before and after *in vitro* thrombolysis. The r-SAK activity grouped by antibody titers was compared using one-way ANOVA, and pairwise comparisons were performed using the least significant difference test method. ^**^P < 0.01. Abbreviations: r-SAK, recombinant staphylokinase; FAPA, fibrin-agarose plate assay; AU, activity unit.

The results of r-SAK activity are shown in ***Fig. 7A***. Consistent with the neutralization experiments, r-SAK activity significantly decreased at the higher antibody titer (2.20) compared with the lower titer (1.90), both before (138.83 [± 117.83] activity unit (AU)/mL *vs.* 41.31 [± 56.28] AU/mL, *P* = 0.006) and after (150.46 [± 143.28] AU/mL *vs.* 38.68 [± 49.80] AU/mL, *P* = 0.003) thrombolysis (***Fig. 7B***). No significant difference in the r-SAK activity was observed before and after thrombolysis at any antibody titer (***Fig. 7B***).

## Discussion

The present study investigated the one-year clinical outcomes of the OPTIMA-5 study and demonstrated that the contemporary facilitated PCI with a single bolus of half-dose r-SAK numerically reduced the risk of one-year MACE. The anti-r-SAK antibody persisted for more than one year, suggesting that a second r-SAK thrombolysis may not be necessary until one year after the first r-SAK thrombolysis, if required.

Previous facilitated PCI trials did not achieve favorable clinical outcomes^[5–16]^, most probably because of the limited efficacy of thrombolytic agents in dissolving thrombus and the high incidence of bleeding complications, which ultimately offset the potential clinical net benefits^[13,15]^. For example, in the Assessment of the Safety and Efficacy of a New Treatment Strategy with Percutaneous Coronary Intervention (ASSENT-4 PCI) trial, the incidence of MACE was significantly higher in the facilitated PCI group, showing more ischemic cardiac complications and a slight excess of major noncerebral bleeding complications^[13]^. Similarly, the Facilitated Intervention with Enhanced Reperfusion Speed to Stop Events (FINESSE) trial found that facilitated PCI did not provide additional benefits over primary PCI in patients with STEMI and was associated with an increased risk of bleeding^[15]^.

Based on the lessons from previous studies, we introduced several key improvements in the OPTIMA-5 study to minimize ischemic and bleeding risk, a strategy we termed "contemporary facilitated PCI"^[4]^. First, we used a third-generation thrombolytic agent, r-SAK, and selected a half-dose regimen to reduce bleeding risk^[17]^. This strategy exhibited high efficacy in rapidly dissolving thrombi without significantly increasing bleeding, highlighting the strong fibrin selectivity and potency of r-SAK^[17–20]^. Second, we adopted a novel P2Y_12_ inhibitor, ticagrelor, for the concomitant antithrombotic therapy. In STEMI patients undergoing fibrinolytic therapy, ticagrelor demonstrated more rapid, potent, and sustained inhibition of platelet aggregation than clopidogrel, while its post-fibrinolytic administration was non-inferior to clopidogrel regarding major bleeding^[21–24]^.

These modifications appeared to improve MACE outcomes during the one-year follow-up. Notably, the difference in MACE incidence between the two groups was most evident within the first three months, mainly driven by the low rates of heart failure and cardiogenic shock. This finding indicated that the OPTIMA-5 strategy enhanced early cardiac recovery after STEMI, likely due to a smaller infarct size, as evidenced by cardiac magnetic resonance findings in the OPTIMA-5 study^[4,25]^.

The present study was the first to investigate the one-year variations in anti-r-SAK antibody levels in STEMI patients who received a bolus of half-dose r-SAK before facilitated PCI. The exploration of the correlation between *in vitro* thrombolysis rate and antibody titers contributed to determining the optimal timing for a second thrombolysis. The most significant decline in antibody titers was observed between levels (lg) of 1.90 and 2.20, with the antibody titer (lg) being 2.19 on day 180 and 1.73 on day 360. Given the similar thrombolysis rates observed between an antibody titer (lg) of 1.90 and the antibody-negative control, a second r-SAK thrombolysis may be considered only after one year following the first r-SAK thrombolysis, if necessary. By detecting r-SAK activity, we observed a significant reduction in activity as the antibody titer (lg) increased from 1.90 to 2.20, both before and after thrombolysis. This indicated that the anti-r-SAK antibody significantly neutralized r-SAK activity once the antibody titer (lg) reached 2.20, consistent with the findings of the *in vitro* thrombolysis study.

Previous studies demonstrated that r-SAK administration did not trigger allergic responses, with specific anti-staphylokinase IgG antibodies emerging in 73% of patients after two weeks^[26]^. Other studies showed that patients developed specific neutralizing IgG antibodies 10–12 days after r-SAK infusion, peaking at 3–9 weeks, and lasting over one year, with lower allergenicity than streptokinase^[27–29]^.

As SAK is a non-human-derived bacterial protein secreted by *Staphylococcus aureus*, anti-r-SAK antibodies may be detected in patients with prior *Staphylococcus aureus* infections, even without previous r-SAK treatment^[17]^. Vakili *et al*^[17]^ illustrated that, although lower levels of anti-r-SAK antibody titers could be detected in healthy individuals, allergic reactions to r-SAK were rare. Similarly, in the present study, lower levels of anti-r-SAK antibody titers (lg) of 0.38 ± 0.67 were observed in the NS group, with a positive rate of 25.6%, and no allergic reactions to r-SAK occurred in the r-SAK group.

The study's findings hold significant clinical implications for the treatment of STEMI patients. First, our strategy demonstrated a numerical reduction in one-year MACE, indicating its potential as an alternative to traditional reperfusion strategies. Second, the exploration of the dynamic changes of anti-r-SAK antibody levels provided critical insights into the optimal timing for r-SAK rethrombolysis, which may guide future clinical practice. Third, larger-scale trials should be conducted to confirm the clinical efficacy of contemporary facilitated PCI, and to explore its long-term benefits.

The present study has several limitations. First, the investigators were not blinded to treatment allocations. However, all endpoints were adjudicated by an independent ERC blinded to these allocations. Second, based on the sample size and incidence of MACEs (14% in the r-SAK group; 20% in the NS group), with a significance level (*α*) set at 0.05, the statistical power was calculated to be 0.21, which is insufficient to confirm the difference between the two groups regarding MACE. However, this pilot study provides important information for our subsequent OPTIMA-6 trial, which aims to investigate the efficacy of contemporary facilitated PCI strategy regarding MACE and major bleeding^[30]^. Third, because of the COVID-19 pandemic, the number of samples collected in the 90-day anti-r-SAK antibody test was limited; however, sufficient samples were collected from 180 days to 360 days to evaluate antibody titers and safety of rethrombolysis. Fourth, the follow-up period was relatively short, and longer-term follow-up data would provide a more comprehensive understanding of the durability of observed benefits.

### Conclusions

For STEMI patients expected to undergo PCI within 120 min, a single bolus of half-dose r-SAK before primary PCI may reduce one-year MACE rates. Following r-SAK thrombolysis, the anti-r-SAK antibody persists for over one year, and a second r-SAK thrombolysis may not be required until one year after the first r-SAK thrombolysis, if necessary.

## SUPPLEMENTARY DATA

Supplementary data to this article can be found online.
