# Supplementary material for: One-year outcomes of a single bolus r-SAK before primary PCI for STEMI: Follow-up of the OPTIMA-5 study
Source: J Biomed Res. 2025 May 21;39(6):611–21. doi: 10.7555/JBR.39.20250043 (PMC12683507; doi:10.7555/JBR.39.20250043)
Supplement: Supplementary file 1 — Supplementary data to this article can be found online. [file jbr-39-6-611-Supplementary.pdf]

Available online at www.jbr-pub.org.cn

Open Access at PubMed Central

Journal of Biomedical Research, 2025 39(6): 1–8

JBR

Supplementary Data

One-year outcomes of a single bolus r-SAK before primary PCI for STEMI: Follow-up of the OPTIMA-5 study

Chen Li[1,△], Jie Yu[1,△], Tian Wu[1,△], Qingxia Lin[1,△], Rui Hua[1,△], Zihang Zhong[2,△], Yule Li[3], Kun Liu[4], Li Zhu[5], Naiquan Yang[6], Xin Chen[7], Xiaoyan Wang[8], Xin Zhao[9], Jun Jiang[10], Bo Zhao[4], Xiwen Zhang[11], Pengsheng Chen[12], Tong Wang[13], Yi Xu[14], Gaoyong Liao[15], Liang Yuan[1], Bo Chen[1], Zhihui Xu[1], Xiaoxuan Gong[1], Wenhao Zhang[1], Chunyue Tan[1], Lei Xu[1], Qiang Huang[1], Jianling Bai[2,✉], John W Eikelboom[16], Chunjian Li[1,✉]

[1]*Department of Cardiology, the First Affiliated Hospital of Nanjing Medical University, Nanjing, Jiangsu 210029, China;*
[2]*Department of Biostatistics, School of Public Health, Nanjing Medical University, Nanjing, Jiangsu 211166, China;*
[3]*College of Letters and Science, University of Wisconsin−Madison, Madison, WI 53715-1007, USA;*
[4]*Department of Cardiology, the First People's Hospital of Lianyungang, Lianyungang, Jiangsu 222002, China;*
[5]*Department of Cardiology, Taizhou People's Hospital, Taizhou, Jiangsu 225300, China;*
[6]*Department of Cardiology, Huai'an Second People's Hospital Affiliated to Xuzhou Medical University, Huai'an, Jiangsu 223200, China;*
[7]*Department of Cardiology, the Affiliated Changzhou No. 2 People's Hospital of Nanjing Medical University, Changzhou, Jiangsu 213164, China;*
[8]*Department of Cardiology, Affiliated Hospital of Jiangnan University, Wuxi, Jiangsu 214000, China;*
[9]*Department of Cardiology, the Second Hospital of Dalian Medical University, Dalian, Liaoning 116027, China;*
[10]*Department of Cardiology, the Second Affiliated Hospital of Zhejiang University School of Medicine, Hangzhou, Zhejiang 310009, China;*
[11]*Department of Cardiology, the Affiliated Huai'an No. 1 People's Hospital of Nanjing Medical University, Huai'an, Jiangsu 223300, China;*
[12]*Department of Cardiology, Xuzhou Central Hospital, Xuzhou, Jiangsu 221000, China;*
[13]*Department of Cardiology, Yancheng No. 1 People's Hospital, Yancheng, Jiangsu 224000, China;*
[14]*Department of Radiology, the First Affiliated Hospital of Nanjing Medical University, Nanjing, Jiangsu 210029, China;*
[15]*Xintrum Pharmaceuticals Co., Ltd., Nanjing, Jiangsu 211100, China;*
[16]*Department of Medicine, McMaster University or Thrombosis Service, Hamilton General Hospital, Hamilton, Ontario L8S 4L8, Canada.*

Supplementary Data 1 Committee member lists

Data and safety monitoring board

An independent Data and Safety Monitoring Board (DSMB) was established to monitor the accruing safety and outcome data. The DSMB is composed of independent cardiologists, independent interventionists, and one independent biostatistician. The DSMB reviews safety data regularly, and conducts analyses of the data at the request of the Steering Committee. All DSMB analyses and operations are formally separated from the sponsor, investigators, and Steering Committee. The DSMB advises the Study Chairman by providing recommendations on trial continuation or other aspects of study conduct. The DSMB members

△These authors contributed equally to this work.

✉Corresponding authors: Chunjian Li, Department of Cardiology, the First Affiliated Hospital of Nanjing Medical University, 300 Guangzhou Road, Nanjing, Jiangsu 210029, China. E-mail: lijay@njmu.edu.cn; Jianling Bai, Department of Biostatistics, School of Public Health, Nanjing Medical University, 101 Longmian Avenue, Nanjing, Jiangsu 211166, China. E-mail: jbai@njmu.edu.cn.

Received: 30 January 2025; Revised: 05 May 2025; Accepted: 08 May 2025; Published online: 21 May 2025

CLC number: R542.22, Document code: A

Chunjian Li received a donation of r-SAK and funds from Kanion Pharmaceutical Group (Lianyungang, China). All other authors declared no conflict of interest.

This is an open access article under the Creative Commons Attribution (CC BY 4.0) license, which permits others to distribute, remix, adapt and build upon this work, for commercial use, provided the original work is properly cited.

© 2025 by Journal of Biomedical Research.

https://doi.org/10.7555/JBR.39.20250043

are listed in ***Supplementary Table 1***. The entire project is overseen by the Steering Committee (SC), whose members are listed in ***Supplementary Table 2***.

Events review committee (ERC)

This study adopts a systematic process for verifying each event by relevant professional experts as follows: (1) If the auditor agrees with the original researcher's event report: The auditor indicates agreement or uncertainty on the event verification report; the event verification database is updated by the verification assistant; the original investigator's judgment of the event is accepted; verification concludes. (2) If the first auditor disagrees with the original researcher's event report: The auditor indicates disagreement on the event verification report; the auditor must report a new classification or diagnosis based on their assessment of the verification data; The event verification data and new report are sent to a second auditor who is blind to the first auditor's judgement, for further review. (3) If the second auditor agrees with the original investigator: The auditor indicates agreement on verification report; the original investigator's judgment is accepted, and the first auditor's opinion is rejected; the event verification database is updated, and verification concluded; verification concludes. (4) If the second auditor disagrees with the original investigator but agrees with the first auditor: The auditor indicates disagreement on the verification report; the original investigator's judgment is rejected, and the first auditor's judgment is accepted; the event verification database is updated, and verification concluded; verification concludes. (5) If the second auditor is uncertain about the classification: The auditor indicates uncertainty on the verification report; the first and second auditors discuss the event to reach a consensus. (6) If both auditors agree with the original investigator's event report: The auditor indicates agreement on the verification report; the original investigator's judgment is accepted; the event verification database is updated, and verification concluded; verification concludes. (7) If both auditors disagree with the original investigator's event report: The auditor indicates disagreement on the verification report; the first auditor's judgment is accepted; the event verification database is updated, and verification concluded; verification concludes. (8) If the two auditors' opinions remain inconsistent: The lack of consensus is documented; the event is escalated to the chair of the verification committee for a final ruling (agreeing or disagreeing with the original researcher's opinion); the event verification database is updated by the verification assistant; verification concludes. The independent ERC members are listed in ***Supplementary Table 3***.

Supplementary Table 1 **Information on Data and Safety Monitoring Board members**

Name	Department	Medical department
Xiuqin Wang	Office of Science and Technology	The First Affiliated Hospital of Nanjing Medical University
Wenxi Wu	General Surgery	The First Affiliated Hospital of Nanjing Medical University
Fuming Zhang	Cardiology	The First Affiliated Hospital of Nanjing Medical University
Ting Wu	Neurology	The First Affiliated Hospital of Nanjing Medical University
Lijuan Chen	Hematology	The First Affiliated Hospital of Nanjing Medical University
Yang Zhao	Epidemiology and Biostatistics	Nanjing Medical University

Supplementary Table 2 **Information on Steering Committee members**

Name	Department	Medical department
John W Eikelboom	Hematology	McMaster University Hamilton General Hospital
Hongbing Shen	Epidemiology and Biostatistics	Chinese Center for Disease Control and Prevention
Jun Zhu	Cardiology	Fuwai Hospital, National Center for Cardiovascular Diseases
Jun Huang	Cardiology	The First Affiliated Hospital of Nanjing Medical University
Xiangqing Kong	Cardiology	The First Affiliated Hospital of Nanjing Medical University
Hao Yu	Epidemiology and Biostatistics	Nanjing Medical University

Supplementary Table 3 **Information on Events Review Committee members**

Name	Department	Medical Department
Jun Zhu	Cardiology	Fuwai Hospital and National Center for Cardiovascular Diseases, Chinese Academy of Medical Sciences and Peking Union Medical College
Xinli Li	Cardiology	The First Affiliated Hospital of Nanjing Medical University
Ting Wu	Neurology	The First Affiliated Hospital of Nanjing Medical University
Jianqing Ge	Neurology	Nanjing Brain Hospital Affiliated with Nanjing Medical University
Chengchun Tang	Interventional Cardiology	Zhong-Da Hospital Affiliated with Southeast University
Jie Song	Interventional Cardiology	Gu-Lou Hospital Affiliated with Nanjing University
Mingfang Li	Cardiology	The First Affiliated Hospital of Nanjing Medical University

Supplementary Data 2 Definitions of study endpoints

Cardiovascular (CV) death[1]

Sudden cardiac death

Death that occurs unexpectedly in a previously stable patient and includes the following deaths: Witnessed and instantaneous without new or worsening symptoms; witnessed within 60 min of the onset of new or worsening cardiac symptoms; witnessed and attributed to an identified arrhythmia (*e.g.*, captured on electrocardiogram [ECG] recording or witnessed on a monitor by either a medic or paramedic); patients unsuccessfully resuscitated from cardiac arrest or successfully resuscitated but who die within 24 h without identification of a non-cardiac etiology; unwitnessed death without conclusive evidence of a non-CV cause (*i.e.*, presumed CV death).

Sudden death due to acute myocardial infarction (MI)

Sudden death occurring within 14 days after a documented acute MI (verified either by the universal definition of MI[2] or by autopsy findings showing recent MI or recent coronary thrombus) and without conclusive evidence of another cause. If death occurs before biochemical confirmation of myocardial necrosis can be obtained, adjudication should rely on clinical presentation and ECG evidence.

Death due to heart failure (HF) or cardiogenic shock

Death occurring in the context of worsening symptoms and/or signs of congestive HF without evidence of another cause. New or worsening signs and/or symptoms of HF include any of the following: (1) New or increasing symptoms and/or signs of HF requiring the initiation or intensification of HF therapy or occurring in a patient already on maximal therapy; (2) HF symptoms or signs requiring continuous intravenous therapy or oxygen administration; (3) Confinement to bed predominantly due to HF symptoms; (4) Pulmonary edema sufficient to cause tachypnea and distress, not related to an acute MI or arrhythmia independently of HF worsening; (5) Cardiogenic shock, not associated with an acute MI or arrhythmia occurring independently of HF worsening.

Cardiogenic shock is defined as systolic pressure (SBP) <90 mmHg for more than 1 h, unresponsive to fluid resuscitation and/or heart rate correction, and attributed to cardiac dysfunction, accompanied by at least one of the following signs of hypoperfusion: cool and clammy skin; oliguria (urine output <30 mL/h); altered sensorium; cardiac index <2.2 L/(min·m^2). Cardiogenic shock may also be defined in the presence of SBP ≥90 mmHg or duration <1 h if blood pressure or duration is influenced by positive inotropic or vasopressor agents, with or without mechanical support.

Death due to stroke, cerebrovascular events

Death occurring within 30 days after a stroke, either directly due to the stroke or a complication of the stroke.

Death due to other CV causes

Death resulting from a fully documented CV cause not included in the categories above such as dysrhythmia, pulmonary embolism, or CV intervention. Death due to an MI occurring as a direct consequence of a CV procedure will be classified under this category. In addition, death due to hemorrhage will be included[3].

Death due to an unknown cause[4]

These are expected to be rare and occur only when no supportive documentation is available, despite repeated attempts to obtain it.

Non-CV death[1]

Non-CV death is defined as any death not classified as cardiac or vascular. Examples include death due to:

pulmonary causes, renal, or gastrointestinal causes, infection (including sepsis), non-infectious conditions, such as systemic inflammatory response syndrome, malignancy (*i.e.*, new onset and progression of prior malignancy), accidental, trauma, suicide, or non-CV organ failure (*e.g.*, hepatic failure) or non-CV surgery complications.

Reinfarction

Criteria for acute MI (types 1, 2, and 3 MI)[2]

The term acute MI should be used when there is evidence of acute myocardial injury accompanied by clinical signs of myocardial ischemia and with detection of a rise and/or fall of cardiac troponin (cTn) values, with at least one value above the 99th percentile upper reference limit (URL) and at least one of the following: (1) Symptoms of myocardial ischemia; (2) New ischemic ECG changes; (3) Development of pathological Q waves; (4) Imaging evidence of new loss of viable myocardium or new regional wall motion abnormality in a pattern consistent with an ischemic etiology; (5) Identification of a coronary thrombus by angiography or autopsy (not for types 2 or 3 MIs).

Post-mortem demonstration of acute atherothrombosis in the artery supplying the infarcted myocardium meets the criteria for type 1 MI. Evidence of an imbalance between myocardial oxygen supply and demand unrelated to acute atherothrombosis meets the criteria for type 2 MI. Cardiac death in patients with symptoms suggestive of myocardial ischemia and presumed new ischemic ECG changes before cTn values become available or abnormal meets the criteria for type 3 MI.

Criteria for coronary procedure-related MI (types 4 and 5 MI)[2]

Criteria for coronary procedure-related MI (types 4 and 5 MI) include percutaneous coronary intervention (PCI)-related MI, termed type 4a MI, and coronary artery bypass grafting (CABG)-related MI, which is termed type 5 MI.

Coronary procedure-related MI ≤48 h after the index procedure is arbitrarily defined by an elevation of cTn values >5 times the 99th percentile URL for type 4a MI and >10 times for type 5 MI in patients with normal baseline values. Patients with elevated pre-procedural cTn values, in whom the pre-procedural cTn level is stable (≤20% variation) or falling, must meet the criteria for a >5 or >10-fold increase and show a >20% change. In addition, at least one of the following must be present: (1) New ischemic ECG changes (applies only to type 4a MI); (2) Development of new pathological Q waves; (3) Imaging evidence of loss of viable myocardium that is presumed to be new and in a pattern consistent with an ischemic etiology; (4) Angiographic findings consistent with a procedural flow-limiting complication such as coronary dissection, occlusion of a major epicardial artery or graft, side-branch occlusion-thrombus, disruption of collateral flow, or distal embolization.

Within the first 24 h after randomization[5]

Reinfarction is defined as new ischemic symptoms lasting more than 20 min and new or recurrent ST-segment elevation or depression >0.1 millivolt in >2 contiguous leads.

Between 24 h and 7 days after randomization[5]

Reinfarction is defined as ischemic symptoms lasting more than 20 min either: re-elevation of cTn to above the upper limit of normal and increased by ≥ 50% over the previous value[6]; or new or recurrent ST-segment elevation or depression >0.1 millivolt or new significant Q-waves in >2 contiguous leads, discrete from the baseline MI.

After 7 days of randomization[5]

Reinfarction is defined according to *Criteria for acute MI (types 1, 2, and 3)* and *Criteria for coronary procedure-related MI (types 4 and 5)*.

Unplanned target vessel revascularization (TVR)[7]

Unplanned TVR is defined as any unexpected repeat percutaneous intervention or surgical bypass of any segment of the target vessel. The target vessel is defined as the entire major coronary vessel proximal and distal to the target lesion, which includes upstream and downstream branches and the target lesion itself.

Heart failure and cardiogenic shock

Heart failure[6,8]

The patient exhibits documented new or worsening symptoms due to HF on presentation, including at least one of the following: (1) Dyspnea (dyspnea with exertion, dyspnea at rest, orthopnea, paroxysmal dyspnea); (2) Decreased exercise tolerance; (3) Fatigue; and (4) Other symptoms of worsened end-organ perfusion such as dizziness, mental confusion or volume overload such as weight gain or lower extremity swelling. The patient also has objective evidence of new or worsening HF, requiring treatment

with diuretics, as shown at least one of the following: (1) Pulmonary edema/congestion on chest X-ray or computed tomography (CT) without suspicion of a non-cardiac cause; (2) Rales >1/3 up from the lung base (Killip class 2 or higher); (3) Pulmonary capillary wedge pressure (PCWP) >25 mmHg; (4) PO_2 <80 mmHg or O_2 sat <90% (no supplemental O_2) in the absence of known lung disease; and (5) Increased B-type natriuretic peptide (BNP)/N-terminal proBNP (NT-proBNP) concentrations consistent with HF decompensation.

Cardiogenic shock[6]

Cardiogenic shock is defined as one of the following: (1) SBP <90 mmHg for at least 30 min (or the need for vasopressor agents and/or mechanical circulatory support to maintain an SBP >90 mmHg) in the presence of a heart rate >60 beats/min, with signs of end-organ hypoperfusion (cold extremities, urinary output <30 mL/h and/or mental confusion); or (2) A cardiac index <2.2 L/(min·m^2) in the presence of PCWP >15 mmHg.

Major ventricular arrhythmia[9]

Ventricular arrhythmias occurring more than six hours after randomization and requiring electrical cardioversion/defibrillation.

Cardiac mechanical complications[10]

Defined as ventricular septal rupture, papillary muscle rupture, cardiac rupture, or ventricular aneurysm diagnosed by echocardiography after acute MI. (1) Ventricular septal rupture: Within 7 days after acute MI; Echocardiography shows shunt flow across ventricular septum and simple apical defect or extensive irregular inferobasal defect; (2) Papillary muscle rupture: Within 7 days after acute MI; Echocardiography shows ruptured papillary muscle, prolapse/flail leaflets, or severe mitral regurgitation; (3) Cardiac rupture: Within 7 days after acute MI; Echocardiography shows pericardial effusion/tamponade or pericardial clots; and (4) Ventricular aneurysm: After acute MI; Echocardiography shows a full-thickness scar that is characterized by a localized, convex protrusion during both phases of the cardiac cycle, and complete loss of contractility as shown by akinesia or paradoxical motion during systole[11].

Stroke[4]

Stroke is defined as the presence of an acute focal neurological deficit thought to be of vascular origin, with signs and symptoms lasting ≥24 h or until the time of death. Based on clinical presentation and CT or magnetic resonance imaging (MRI) findings, strokes will be classified as follows.

Definite ischemic stroke

Stroke with CT or MRI performed within 3 weeks that show no evidence of primary hemorrhage (hemorrhagic transformation is consistent with ischemic stroke).

Definite hemorrhagic stroke

(1) Primary intracerebral, intraparenchymal, or intraventricular brain hemorrhage: Stroke with CT or MRI evidence of acute cerebral hemorrhage. Note: Does not include hemorrhage secondary to cerebral infarct, post-traumatic intracerebral hemorrhage, hemorrhage into a tumor, or hemorrhage into a vascular malformation. (2) Subarachnoid hemorrhage: Typical clinical presentation of sudden onset headache, with or without focal signs (subarachnoid hemorrhage may not have focal deficit), and CT or cerebrospinal fluid evidence of bleeding primarily in the subarachnoid space. Non-traumatic subarachnoid hemorrhage documented by imaging is considered a hemorrhagic stroke.

Uncertain or unknown stroke

Definite stroke that does not meet the above criteria for cerebral infarction or hemorrhage (CT scan or MRI not done). The neurological deficit must have been present for 24 h. NOTE: Subdural and epidural hematomas are not considered strokes but will be classified as major hemorrhages.

Supplementary Data 3 Detection of the recombinant staphylokinase (anti-r-SAK) antibody

Method

This analytical method includes screening experiments, immunoinhibition (confirmatory) experiments, and titer detection experiments. The screening experiment is used to detect potential anti-drug antibodies (ADAs) in serum samples. When the response value (S/N) is equal to or greater than the screening threshold, the sample must undergo an immunoinhibition confirmation experiment. The confirmation experiment is based on the principle that a high concentration of free drug competitively inhibits the specific binding of drug-resistant antibodies to the immobilized (coated) drug. The data obtained from the samples with or without (drug

treatment) are compared with the confirmation threshold to determine the final result of the sample.

Titer measurements are performed when the sample is confirmed to be positive and the response value in the screening experiment meets or exceeds the titer cut point (TCP). The titer results are reported as follows: Samples confirmed positive, with signal values not lower than TCP in the screening experiment, are reported as the product of the maximum dilution of the sample and the minimum required dilution (MRD), and samples confirmed positive but with signal values lower than TCP are reported as less than MRD, and titer measurement is not required.

This analysis method is based on the enzyme-linked immunosorbent assay (ELISA). The principle is as follows: firstly, the sample is added to a 96-well removable plate pre-coated with the test drug and incubated at room temperature with shaking, allowing ADAs in the sample to form an ADA-drug complex on the solid phase. After washing, the detection reagent r-SAK is added and the plate is again incubated at room temperature. Finally, the TMB is added after washing, and the instrument signal value is proportional to the concentration of the antibody. Positive control samples are prepared in 100% normal healthy human serum.

Reagents, materials, and instruments

Controls, key reagents, and matrices are listed in ***Supplementary Table 4***. Test solution and reagent are listed in ***Supplementary Table 5***. Main instruments are listed in ***Supplementary Table 6***.

System suitability sample and analysis method parameter

The analytical run contains three sets of negative control samples (NC) with a final concentration of 2 μg/mL for the test drug r-SAK. The procedure for sample testing is shown in ***Supplementary Fig. 1***.

Data processing

The instrument response values recorded using SpectraMax I3X control software (SoftMax Pro 7.0.2S), and the raw data were analyzed according to the specifications of the analytic protocol. Microsoft Excel 2010 (Microsoft Office 2010) was used for further data processing.

Operation process

The operation procedure includes following steps: (1) Coating: Dilute r-SAK to the target concentration with coating buffer one day before the formal experiment. Add 100 μL per well to a high-binding 96-well plate, seal with a microplate sealer, and store at 2–8 ℃ overnight (18–20 h). (2) Washing: Remove the sealed plate, discard the liquid, wash with washing solution buffer using the 3 T program, and blot residual liquid on absorbent paper. (3) Blocking: Add 300 μL of 1% BSA blocking solution per well, seal the plate, and incubate at room temperature for approximately 2.5 h.

Supplementary Table 4 **Information on controls, key reagents, and matrices**

Name	Item number	Lot number	Factory
Recombinant staphylokinase	–	–	Jiangsu Zhongxin Pharmaceutical Co., Ltd.
96-Well enzyme plate	42 592	34 520 012	COSTAR®
ZX2020-Biotin	–	20 220 727	Jiangsu Zhongxin Pharmaceutical Co., Ltd.
Polyclonal antibody (rabbit)	–	RD2202180751	GenScript
Streptavidin-HRP	S2438-250UG	SLCJ3738	Sigma
Blank human serum	–	–	Procurement by Jiangsu Zhongxin Pharmaceutical Co., Ltd.

Supplementary Table 5 **Information on test solution and reagent**

Name	Item number	Source
Diluent/blocking solution: 1% BSA in PBS	–	Jiangsu Zhongxin Pharmaceutical Co., Ltd.
Plate washing solution: 1*PBST	–	Jiangsu Zhongxin Pharmaceutical Co., Ltd.
Acetic acid (acetic acid)	695 092	Sigma
1M Tris	–	Jiangsu Zhongxin Pharmaceutical Co., Ltd.
Stop solution: 2M sulfuric acid	–	Jiangsu Zhongxin Pharmaceutical Co., Ltd.
TMB	P0209	Biyuntian

Supplementary Table 6 **Information on main instruments**

Name	Model	Factory
Board reader	SpectraMax I3X	Molecular Device Inc.
Plate washer	Wellwash	Thermo Inc.
Pipette	Eppendorf	Eppendorf Inc.
Ultra-low temperature freezer (−60–−90 ℃)	Forma 900	Thermo Inc.
Low temperature freezer (−10–−30 ℃)	YCD-EL259A	Zhongke Meiling Low Temperature Technology Co., Ltd.
Refrigerated refrigerator (2–8 ℃)	YC-968L	Zhongke Meiling Low Temperature Technology Co., Ltd.
Thermostatic oscillator	HN70-2A	Shanghai Khanno Instrument
Microplates/enzyme plates	Costar 42 592, 96 wells	COSTAR, USA
Microcentrifuge tubes	Polypropylene centrifuge tubes	Fisher Scientific
Pointed	–	KIRGEN

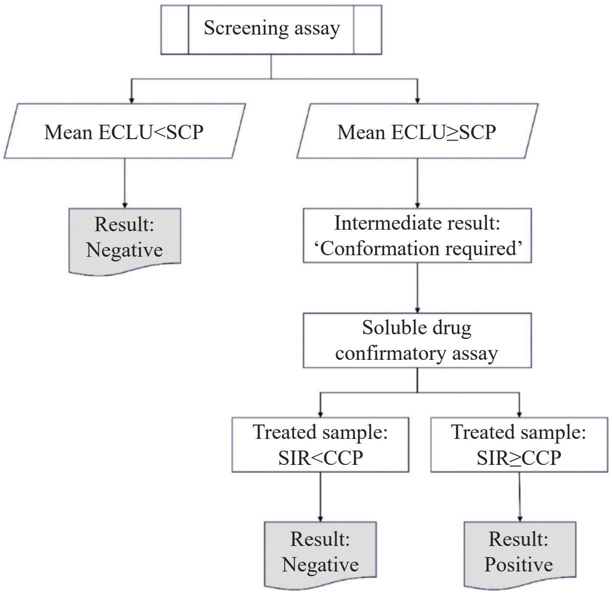

Supplementary Fig. 1 **Flowchart for defining the positive cut-off of anti-r-SAK antibody titers.** Abbreviations: CCP, confirmatory cut point; ECLU, enzyme-linked chemiluminescent units; SCP, screening cut point; SIR, signal inhibition ratio.

(4) Acidification: During the blocking step, dilute the positive control antibody to the target concentration with a mixed blank matrix. Dilute the serum samples with 300 mmol/L ice-cold acetic acid in a low-binding dilution plate and incubate at 25 ℃, 700 rpm for 40 min. (5) Washing: Remove the blocking plate, discard the liquid, wash using the 3 T program as follows: add 300 μL washing solution per well, shake gently for 30 s, and allow to stand for 1 to 2 min; vigorously discard solution and blot the plate several times on absorbent paper until no liquid remains; repeat this process three times. (6) Sample addition: Add 15 μL of 1 mol/mL Tris solution to each well, then add 50 μL of Biotin solution, and finally add 42 μL of acidified sample. Seal the plate with a microplate sealer and incubate at 25 ℃, 450 rpm for 2 h. (7) Washing: Wash the plate again using the 3T program and blot dry as described above. (8) Secondary antibody addition: Add 100 μL of secondary antibody solution to each well of the plate and incubate at 25 ℃, 450 rpm for 1 h. (9) Washing: Discard the liquid and wash again using the 3T program, then blot dry on absorbent paper. (10) Color development: Add 100 μL of TMB color liquid to each well of the plate, incubate at 25 ℃, avoid light, and incubate at 450 rpm for 10–15 min. (11) Reaction termination: Add 100 μL of stop solution to each well, and measure the OD value at 450 nm on a microplate reader within 30 min. (12) Reading: Measure the absorbance at a wavelength of 450–620 nm using a plate reader, with a shaking duration of 5 s before reading.

Supplementary Data 4 Detection of the r-SAK activity

The fibrin plate assay relies on the formation of a complex between a plasminogen and plasminogen activator, which activates free plasminogen into biologically active plasmin. Plasmin degrades human fibrin into soluble fibrin fragments, resulting in the appearance of transparent dissolution rings on a fibrin plate. This method was used to quantitatively measure the biological activity of r-SAK.

Reagent preparation

The processes of reagent preparation are as follows: (1) Human Fibrinogen Solution: Prepare with normal saline to a concentration of 6 mg/mL. Stored at −20 ℃. (2) Human Plasminogen Solution: Prepare with normal saline to a concentration of 0.5 mg/mL. Stored at −20 ℃. (3) Human Thrombin Solution: Prepare with normal saline to a concentration of

100 IU/mL. Stored at −20 ℃. (4) 0.25% bovine serum albumin (BSA) solution: Weigh 0.5 g of BSA, add normal saline to a volume of 200 mL, and dissolve to obtain the solution. (5) Standard solution: Dilute r-SAK with 0.25% BSA solution to 7.813 AU/mL, 15.625 AU/mL, 31.25 AU/mL, 62.5 AU/mL, 125 AU/mL, and 250 AU/mL, respectively. (6) Test substance solution: Reconstitute samples with the indicated amount of 0.25% BSA solution, and then dilute it with 0.25% BSA solution to 50 AU/mL or 1 μg/mL.

Operation process

The processes of reagent preparation are as follows: (1) Agarose preparation: Weigh 125 mg of agarose and add 23 mL of normal saline. Boil it until the agarose is fully dissolved and swells, forming an agar. Equilibrate the agar in a 56 ℃ water bath. (2) Fibrin plate mixture: Sequentially add 14 μL of human thrombin solution (100 IU/mL), 280 μL human plasminogen solution (0.5 mg/mL), while shaking gently. And then add 2.2 mL of human fibrinogen solution (6 mg/mL) to the agar, mix thoroughly, and ensure continuous shaking until the solution in the test tube appears cloudy. (3) Plate casting: Immediately pour the turbid mixture into a flat dish with a diameter of 9 cm, level it, and let it fully solidify. Place it in a refrigerator at 4 ℃ for more than 30 min (to be used within 2 days). (4) Sample preparation: Dilute the sample with sterilized saline to attain protein content and electrophoretic purity within the standard unit range (calculated as 5 × 10^4 activity unit [AU]/mg). (5) Well Formation: Use a 2 mm hole punch to make holes in the fibrin plate according to the sample quantity. (6) Sample loading: Sequentially add the diluted standard solution and sample to the holes from the outer ring to the inner ring, with 6 μL/well. (7) Incubation: Cover the flat dish and place it in a constant temperature incubator set at 30–35 ℃ for 16 h. (8) Measurement: Measure the diameter of the lysed circles longitudinally and transversely with a vernier caliper, each measurement performed two times and take the average value. (9) Data analysis: Perform a linear regression between the logarithm of the dilution activity of the standard solution and the corresponding lysed circle diameter, obtain the linear regression equation and calculate the activity of the sample based on the measured lysed circle diameter.

References

[1] Sarafidis PA, Tsapas A. Empagliflozin, cardiovascular outcomes, and mortality in type 2 diabetes[J]. *N Engl J Med*, 2016, 374(11): 1092–1094.

[2] Thygesen K, Alpert JS, Jaffe AS, et al. Fourth universal definition of myocardial infarction (2018)[J]. *Circulation*, 2018, 138(20): e618–e651.

[3] Harloff A, Schlachetzki F. Rivaroxaban for stroke prevention after embolic stroke of undetermined source[J]. *N Engl J Med*, 2018, 379(10): 986–987.

[4] Connolly SJ, Eikelboom JW, Bosch J, et al. Rivaroxaban with or without aspirin in patients with stable coronary artery disease: An international, randomised, double-blind, placebo-controlled trial[J]. *Lancet*, 2018, 391(10117): 205–218.

[5] Mehta SR, Granger CB, Boden WE, et al. Early versus delayed invasive intervention in acute coronary syndromes[J]. *N Engl J Med*, 2009, 360(21): 2165–2175.

[6] Armstrong PW, Gershlick AH, Goldstein P, et al. Fibrinolysis or primary PCI in ST-segment elevation myocardial infarction[J]. *N Engl J Med*, 2013, 368(15): 1379–1387.

[7] Cutlip DE, Windecker S, Mehran R, et al. Clinical end points in coronary stent trials: A case for standardized definitions[J]. *Circulation*, 2007, 115(17): 2344–2351.

[8] Packer M, Anker SD, Butler J, et al. Cardiovascular and renal outcomes with empagliflozin in heart failure[J]. *N Engl J Med*, 2020, 383(15): 1413–1424.

[9] Armstrong PW, WEST Steering Committee. A comparison of pharmacologic therapy with/without timely coronary intervention *vs*. primary percutaneous intervention early after ST-elevation myocardial infarction: The WEST (Which Early ST-elevation myocardial infarction Therapy) study[J]. *Eur Heart J*, 2006, 27(13): 1530–1538.

[10] Gong FF, Vaitenas I, Malaisrie SC, et al. Mechanical complications of acute myocardial infarction: A review[J]. *JAMA Cardiol*, 2021, 6(3): 341–349.

[11] Arvan S, Varat MA. Persistent ST-segment elevation and left ventricular wall abnormalities: A 2-dimensional echocardiographic study[J]. *Am J Cardiol*, 1984, 53(11): 1542–1546.
